# Demographic and psychometric predictors associated with engagement in risk-associated alternative healthcare behaviours

**DOI:** 10.1371/journal.pone.0291016

**Published:** 2023-09-21

**Authors:** Bernie Garrett, Timothy Caulfield, Richard Musoke, Blake Murdoch, Xuyan Tang, Joyce S. T. Lam

**Affiliations:** 1 School of Nursing, University of British Columbia, Vancouver, British Columbia, Canada; 2 Health Law Institute, University of Alberta, Edmonton, Alberta, Canada; 3 School of Population and Public Health, University of British Columbia, Vancouver, British Columbia, Canada; 4 Department of Educational and Counselling Psychology, and Special Education, University of British Columbia, Vancouver, British Columbia, Canada; 5 Pacific Parkinson’s Research Centre, Djavad Mowafaghian Centre for Brain Health, University of British Columbia, Vancouver, British Columbia, Canada; University of Buea, CAMEROON

## Abstract

This paper builds on prior work exploring the use of risk-associated alternative healthcare (RAAH) in Canada. RAAH uptake was surveyed to explore the characteristics of adult RAAH users and the value of established psychometric instruments previously used in alternative healthcare studies in predicting RAAH behaviours: the Control Beliefs Inventory (CBI), the Reward Responsiveness Behavioural Activation System (RBAS) scale, the Positive Attitudes to Science (PAS) scale, the Satisfaction with Orthodox Medicine (SOM) scale, and the brief version of the Susceptibility to Persuasion-II (StP-II-B) scale. Findings suggest RAAH is influenced by gender, age, income, education, employment, chronic illness status, and ethnicity. Engagement in some form of RAAH was common (around 40%) and the most common types of RAAH use reported were physical manipulation and herbal/nutritional supplement use. Other higher-risk AH activities (such as use of toxins and physically invasive procedures) were also reported by about 5% of respondents. The StP-II-B and PAS instruments were predictive of the likelihood of engagement in RAAH behaviours, as illustrated by higher risk tolerance, desire for novelty, positive attitude to advertising and social influence, and positive beliefs about science. The CBI, RBAS, and SOM instruments were not predictive overall. However, the CBI and SOM instruments were predictive of engagement with physical manipulative RAAH activities, while the RBAS was predictive of herbal/nutritional RAAH engagement. These findings can help inform health professionals’ understanding of public health-seeking behaviours with respect to risk.

## Introduction

Alternative healthcare (AH) therapies are a range of therapeutics that largely originate from traditions and theories distinct from contemporary biomedical science, and which claim mechanisms of action outside of those currently accepted by scientific and biomedical consensus [1–4]. They often focus on “holistic” personal wellbeing and exist predominantly outside of public healthcare, and many are argued to be beyond the scope of scientific analysis. Some AH therapies are also claimed to supplement conventional biomedical healthcare, such as meditation or acupuncture for anxiety and pain management, and are often collectively referred to as complementary and alternative medicine or integrative medicine [5]. Many AH practices are difficult to validate in empirical terms, and thus there is frequently an adversarial stance between biomedical and AH practitioners [6, 7]. Nevertheless, the use of AH has risen over the past two decades, instigating research into the possible factors associated with its use [4, 8, 9].

Despite many AH practices being physically harmless, some do entail significant risks of adverse events [2, 10, 11]. Research suggests that people are now more frequently engaging in AH practices that involve risk, such as utilizing unproven therapeutics instead of medically established ones [2, 11, 12]. This trend has significant health policy and practice implications. Initial work suggests that socio-demographic factors, beliefs about control of personal health, motivation, susceptibility to persuasion, beliefs about science, and satisfaction with orthodox medicine may help predict those who are most likely to engage in such risk-associated alternative health (RAAH) behaviours. However, this remains a relatively unexplored area. Therefore, building on prior work, the goals of this study were to identify the socio-demographic and psychosocial factors associated with engagement in RAAH, and to establish the factors that may predict engagement behaviours. The key research questions were:

1. What types of RAAH behaviours are most evident in the Canadian public?
2. Can socio-demographic factors and established psychometric tools associated with engagement in AH help predict the likelihood of engagement in RAAH?

### Alternative healthcare and risk

The regulation of AH varies across Canada, and a recent study identified four major categories of RAAH behaviours: general RAAH practices that conflict with the patient’s biomedical care (e.g., using AH instead of recommended medical treatment, people failing to inform their physician of use of alternative therapies concurrently with medical treatment, or using untested alternative therapies), specific RAAH therapies based on specific alternative belief systems (e.g., naturopathic intravenous therapy or cupping), physical manipulative RAAH therapies (e.g., forceful chiropractic spinal or cervical manipulative procedures), and RAAH practices involving known hazardous herbal or nutritional supplements [2]. Although rare, significant adverse events occur from these practices, including major physical injuries and even death, and in all cases the current evidence of efficacy does not appear to support the level of risk in engaging with these therapies. For instance, research has confirmed that many patients use AH remedies, such as *Aristolochia* root that may be toxic or cause drug interactions. Herbal treatments have also been associated with adverse events through direct toxicity, unwanted drug interactions, and psychological harm. Additionally, injuries through physical manipulative AH therapies have been documented [13–19]. A range of AH therapies is also frequently employed as a substitute for medical treatment [20, 21]. One 2002 systematic review of AH in older people identified that elderly patients frequently suffered harm as a result of undertaking AH therapies [22], indicating that this vulnerable population may be particularly at risk.

### Psychological factors influencing alternative healthcare uptake and associated instruments

Psychological theory has suggested a number of psychological factors related to AH use. For instance, belief in personal control of one’s own health has been identified as an important factor in coping with illness and AH use. Decision making related to the use of AH has been seen as one means of regaining control during experiences of uncertainty associated with cancer [23]. AH users have been found to report a higher sense of control over their health, and they use AH to mitigate unpleasant aspects of conventional treatments [4, 9, 23–25]. The idea that patients use AH as it allows them to take more active control in managing health is supported by a number of clinical studies [4, 23, 26–29]. The Control Beliefs Inventory (CBI) is a 26-item psychometric tool developed to assess this characteristic [9]. This self-report measure has been validated in several chronic illness samples independent of health status [29].

Positive motivation is another behavioural factor identified in AH users. It is postulated that two general motivational systems underlie behaviour and affect: a behavioural inhibition system (BIS) and a behavioural activation system (BAS) [30, 31]. The BIS regulates aversive motives to avoid something unpleasant, whereas the BAS regulates appetitive motives and movement towards something desired. The persistent tendency to seek out or to gain positive rewards (rather than to avoid unpleasant circumstances) has been identified as the predominant motivational system in several AH studies [4, 29, 32–34]. The 5-item Reward Responsiveness BAS scale (RBAS) was developed and validated as a tool useful in assessing this phenomenon in AH users. It is a component of Carver and White’s BIS/BAS instrument, and is scored on a 4-point Likert-type scale (agree a lot to disagree a lot) with good internal reliability as indicated by a Cronbach’s α of 0.73 [29, 30].

Studies have also identified that there are also some specific negative drivers, notably, negative personal beliefs about the value of science and dissatisfaction with orthodox medicine, that stimulate motivation away from conventional medicine [8, 35, 36]. Survey tools to explore these drivers have been developed and tested by these researchers, such as the 4-item Positive Attitudes to Science (PAS) and the 6-item Satisfaction with Orthodox Medicine (SOM) surveys [8, 36].

Lastly, research suggests that as with other consumer health products and services, the marketing of AH employs similar techniques of persuasion and often uses those seen in deceptive advertising or health scams [7, 37–40]. Social psychologists have established the mechanisms that affect perception of risk and compliance in high-risk marketing practices [41], and persuasion and influence techniques were highly significant. Ten personality traits have been identified that correlate well with a likelihood of engagement with risky products or services: a dislike of premeditation, liking consistency (not liking changing one’s mind), novelty, needing self-control, valuing social influence, similarity (preference for popular products/services), an openness to taking risks, positive attitudes towards advertising, and a need for cognition and for uniqueness [42–44]. The brief version of the Susceptibility to Persuasion-II (StP-II-B) scale is a valid and reliable modular psychometric tool that measures general susceptibility to these techniques using a 30-item, 7-point Likert-type scale—strongly agree to strongly disagree [45]. Items are divided into ten subscales corresponding to these established persuasive factors. This scale has been demonstrated to be predictive of scam compliance, and these persuasion factors also help inform engagement with advertising in RAAH [38, 44].

Overall, these established psychometric tools are useful predictors of AH uptake, but studies assessing their value in predicting engagement with RAAH have not been undertaken. This study was designed to explore the value of these psychometric instruments in predicting RAAH behaviours.

## Materials and methods

This work builds on prior work that established a taxonomy of RAAH practices [2]. A web-based survey of public engagement in AH practices in Canada incorporating five psychometric tools (CBI, RBAS, PAS, SOM, and StP-II-B) that are correlated with AH uptake was undertaken. Basic demographic details were collected along with attitudinal questions incorporating the psychometric tools and an open-ended question inviting comments from participants: Any feedback on the survey or comments you wish to add? The survey was developed in English and was also translated into French by a certified translator.

### Hypotheses

The researchers sought to test the following two null hypotheses:

1. People who engage in RAAH will demonstrate CBI, RBAS, SOM, PAS, and StP-II-B outcome scores no different than those who do not engage in RAAH.
2. People who engage in more RAAH will demonstrate CBI, RBAS, SOM, PAS, and StP-II-B outcome scores no different than those who engage in them less.

### Sample

Members of the public in Canada aged 16 and over were recruited through two commercial survey providers, Lucid and Amazon Mechanical Turk. Such survey companies are now increasingly employed in social science research due to their convenience and ability to reach a diverse population and balance responses from specific groups [46]. Additional recruitment through Twitter was also adopted with advertisements in health science and AH accounts. As an incentive to complete the survey, each participant had the opportunity to enter into a draw for two $200 Amazon gift vouchers. Personal identifiers used by those participating in the draw were removed from the survey data before analysis to ensure survey anonymity. To achieve an adequate sample size for logistic regression, the recommended rule of thumb is *n* = 100 + 50*i*, where *i* is the number of predictors in the model [47]. As the models used in the present study include 14 predictors, a minimum sample size of 800 was required to obtain accurate estimates.

### Survey

An online cross‐sectional survey was developed using Qualtrics XM, an online survey platform, and was initially pilot-tested with a group of 100 university students in 2020. Following revisions, the survey was administered to the public between October 2021 and April 2022. The survey (available in S1 File) consisted of three parts and was anonymous. The first part requested demographic information as well as a history of participant’s personal experiences of engaging in AH practices. The second part included questions exploring the types of RAAH practices they had engaged in (as identified in earlier work [2]. The third and final part of the survey incorporated questions from the five psychometric tools previously described to specifically explore possible psychological factors that are theorized to influence AH uptake: CBI, RBAS, SOM, PAS, and StP-II-B. The internal consistency reliability of these five measures was established using Cronbach’s α (Table 1) and a value of 0.70 is widely considered good for psychological instruments [48]. All scales and subscales used in this study showed good reliability (Cronbach’s α of ≥ 0.75) except for the PAS, which showed a relatively low but acceptable Cronbach’s α of 0.66 [48].

**Table 1:** Internal consistency reliability estimates of instruments.

Scales/Subscales	Item Number	Cronbach’s α
StP‐II-B Full	30	0.90
Premeditation	3	0.85
Consistency	3	0.83
Sensation seeking	3	0.75
Self-control	3	0.80
Social influence	3	0.90
Similarity	3	0.90
Risk preferences	3	0.95
Attitudes towards advertising	3	0.86
Need for cognition	3	0.85
Preference for uniqueness	3	0.87
CBI Full	26	0.84
Mastery Self-efficacy-CBI	8	0.79
General Control-CBI	7	0.89
Chance Control-CBI	5	0.84
Symptom Control-CBI	6	0.87
RBAS	5	0.84
PAS	4	0.66
SOM	6	0.89

The survey also included an open-ended question exploring participant commentary on their use of AH. Additional simple mathematical calculation and attention-checking questions as well as ballot box stuffing and automated survey checks were included to screen out random and automated responses [49]. The online survey was only accessible to Canadians who reported to be at least 16 years of age.

Ethical approval was obtained from the University of British Columbia’s Behavioural Research Ethics Board (# H19-01790) before data collection, and all participants provided online written informed consent. At the start of the survey, participants were informed the data was anonymous and submission of the survey indicated their consent to participate, and this was reiterated at the end of the survey prior to submission.

### Analysis

Survey responses were exported from Qualtrics to STATA and RStudio for data quality checking, cleaning, and analysis. After removing invalid and incomplete responses, descriptive analyses on the remaining data were conducted to establish the percentage of respondents engaging in AH practices overall and to explore demographic characteristics. Using participants’ responses to questions on engagement in AH practices, binary outcome variables for engagement with any AH were generated (0 = none, 1 = engagement). A second set of outcome variables were generated based on counts of the reported experiences of engaging with the different forms of RAAH practices for both overall and the four RAAH sub-categories (general RAAH, alternative belief systems RAAH, physical manipulative RAAH, and herbal/nutritional RAAH). Logistic regression models were then applied to obtain odds ratios of participants’ experiences of engagement with RAAH practices for each demographic factor and psychometric scale. Rootograms offer an improved approach to the assessment of fit in count regression models [50]. Using rootograms to compare observed and expected values graphically for the count outcomes, zero-inflated negative binomial (ZINB) regression models were found to fit the data better than other models used for count outcomes. Therefore, ZINB models were used to determine the associations between the RAAH categories, together with the demographic factors and psychometric scales. A ZINB regression analysis models two separate processes to produce two sets of coefficients: one for the count outcome, which is the count part of the model, and the other for the binary outcome which is the logistic part of the model. Unlike logistic regression for hypothesis testing, the logistic portion of the ZINB reports the odds of not engaging in the behaviour. For both logistic and ZINB models, bivariate analyses were conducted, and factors found to hold an association (p < 0.25) or conceptually important were added into multivariate regression models. Using backward elimination, factors that were not statistically significant in the full model were dropped and the full model compared to reduced models using likelihood ratio tests and nested Voung test [51] for logistic regression and ZINB, respectively, to obtain parsimonious final models. Reduced and full models were further compared using the Akaike information criteria (AIC) and Bayesian information criteria (BIC) to determine if the reduced models did not increase the AIC and BIC values. Models were built for overall engagement in AH practices and by RAAH category. All statistical analyses were conducted using RStudio version 4.2.1.

## Results

### Sample characteristics

A total of 2253 respondents completed the survey and 761 surveys that had missing data and/or failed data quality checks were removed from the analysis leaving a total sample of 1492 respondents (Table 2). Most of the sample identified as women (58.6%) and Caucasian (66.9%) and reported undertaking some form of paid employment (69.8%), being generally healthy (67.4%), having no chronic illness (58.2%) as well as working outside of the healthcare field (84.7%). Slightly less than half of the sample were middle-aged adults (45.4%), earning an annual income of $49,999 or below (43.7%), and having an education level at the bachelor-level and above (48.3%). Sample characteristics and the mean scores of each psychometric measure across different demographic groups are provided in Table 2.

**Table 2:** Demographic characteristics of respondents with mean and standard deviations of psychometric scores for item averages of each instrument.

		Number of Participants (%)	Number Engaged in RAAH (%)*	CBI (SD)	StP-II-B Mean (SD)	RBAS Mean (SD)	PAS Mean (SD)	SOM Mean (SD)
**Age**								
	34 and below	518 (34.7)	211 (40.7)	4.2 (0.5)	3.9 (0.7)	1.5 (0.5)	5.0 (1.0)	4.8 (1.2)
	35–54	677 (45.4)	317 (46.8)	4.2 (0.5)	3.7 (0.7)	1.6 (0.5)	5.1 (1.0)	5.0 (1.1)
	55 years and above	297 (19.9)	94 (31.6)	4.2 (0.5)	3.5 (0.6)	1.7 (0.5)	5.2 (0.9)	5.3 (1.1)
**Gender**								
	Man	593 (39.7)	212 (35.8)	4.2 (0.5)	3.9 (0.6)	1.7 (0.5)	5.1 (1.0)	4.9 (1.2)
	Woman	875 (58.6)	398 (45.5)	4.2 (0.5)	3.6 (0.7)	1.6 (0.5)	5.0 (0.9)	5.0 (1.1)
	Other†	24 (1.6)	12 (50.0)	3.6 (0.4)	3.6 (0.5)	1.7 (0.5)	5.2 (1.2)	4.5 (1.4)
**Ethnicity**								
	Caucasian	998 (66.9)	444 (44.5)	4.2 (0.5)	3.6 (0.6)	1.6 (0.5)	5.1 (1.0)	5.0 (1.2)
	Asian	222 (14.9)	66 (29.7)	4.2 (0.5)	4.0 (0.6)	1.7 (0.6)	5.1 (0.9)	4.8 (1.0)
	Other^a^	272 (18.2)	112 (41.2)	4.2 (0.5)	3.9 (0.7)	1.6 (0.5)	4.9 (1.0)	4.8 (1.1)
**Education**								
	High school	272 (18.2)	97 (35.7)	4.1 (0.5)	3.9 (0.6)	1.6 (0.6)	4.9 (1.0)	4.7 (1.3)
	College^b^	500 (33.5)	205 (41.0)	4.2 (0.5)	3.7 (0.6)	1.6 (0.5)	5.0 (1.0)	4.9 (1.2)
	Bachelor and above	720 (48.3)	320 (44.4)	4.2 (0.5)	3.6 (0.7)	1.6 (0.5)	5.2 (0.9)	5.1 (1.1)
**Employment**								
	Paid employment^c^	1042 (69.8)	464 (44.5)	4.2 (0.5)	3.8 (0.7)	1.6 (0.5)	5.1 (1.0)	4.9 (1.2)
	No paid employment	450 (30.2)	158 (35.1)	4.1 (0.5)	3.6 (0.6)	1.6 (0.5)	5.1 (0.9)	5.0 (1.2)
**Income**								
	Below $10,000	107 (7.2)	36 (33.6)	4.0 (0.5)	3.7 (0.6)	1.6 (0.5)	4.8 (1.0)	4.7 (1.1)
	$10,000–24,999	186 (12.5)	76 (40.9)	4.1 (0.6)	3.8 (0.6)	1.5 (0.5)	5.1 (1.0)	4.8 (1.3)
	$25,000–49,999	358 (24.0)	126 (35.2)	4.2 (0.5)	3.8 (0.7)	1.6 (0.5)	5.0 (0.9)	4.9 (1.2)
	$50,000–74,999	301 (20.2)	138 (45.8)	4.2 (0.4)	3.7 (0.7)	1.6 (0.5)	5.1 (1.0)	4.9 (1.2)
	$75,000–99,999	242 (16.2)	97 (40.1)	4.2 (0.5)	3.7 (0.6)	1.6 (0.5)	5.1 (0.9)	5.0 (1.1)
	$100,000–124,999	153 (10.3)	79 (51.6)	4.2 (0.5)	3.6 (0.6)	1.6 (0.5)	5.2 (0.9)	5.2 (1.0)
	$125,000–149,999	70 (4.7)	33 (47.1)	4.2 (0.5)	3.7 (0.8)	1.6 (0.4)	5.2 (0.9)	5.2 (1.0)
	$150,000 or more	75 (5.0)	37 (49.3)	4.3 (0.6)	3.7 (0.8)	1.6 (0.5)	5.2 (1.2)	5.1 (1.3)
**Health Status**								
	Frequently unwell	154 (10.3)	81 (52.6)	3.8 (0.5)	3.6 (0.6)	1.6 (0.6)	5.1 (1.1)	4.9 (1.3)
	Generally healthy	1006 (67.4)	406 (40.4)	4.2 (0.4)	3.7 (0.6)	1.6 (0.5)	5.0 (0.9)	4.9 (1.1)
	Very healthy	332 (22.3)	135 (40.7)	4.4 (0.5)	3.8 (0.8)	1.6 (0.6)	5.2 (1.0)	5.1 (1.2)
**Chronic Illness**								
	Yes	623 (41.8)	341 (54.7)	4.1 (0.5)	3.6 (0.7)	1.6 (0.5)	5.1 (1.0)	5.0 (1.2)
	No	869 (58.2)	281 (32.3)	4.3 (0.5)	3.8 (0.6)	1.6 (0.5)	5.1 (0.9)	4.9 (1.1)
**Healthcare Professional**								
	Works in healthcare field	229 (15.3)	128 (55.9)	4.1 (0.5)	3.7 (0.8)	1.6 (0.5)	5.0 (1.0)	5.0 (1.2)
	Does not work in healthcare field	1263 (84.7)	494 (39.1)	4.2 (0.5)	3.7 (0.6)	1.6 (0.5)	5.1 (0.9)	5.0 (1.2)
**Total**		**1492 (100.0)**	**622 (41.7)**	**4.2 (0.5)**	**3.7 (0.7)**	**1.6 (0.5)**	**5.1 (1.0)**	**5.0 (1.2)**

* Row percentages.

† other includes non-binary, preferred not to reveal gender, preferred to self-describe.

a includes Aboriginal, Black, Hispanic, and those who preferred not to mention their ethnicity or any other ethnic group.

b includes participants who reported some college credits, trade, technical, vocational, or associate degrees.

c includes those who reported self-employment, fulltime and part time employment.

### Types of risk-associated alternative health behaviours reported

Participants were asked if they had engaged in specific RAAH activities categorized under four previously established categories of RAAH (general RAAH, alternative belief systems RAAH, physical manipulative RAAH, and herbal/nutritional RAAH) [2]. As shown in Table 3, RAAH uptake was substantial, with 41.7% of respondents reporting they had engaged in at least one form of RAAH activity, and both English and French respondents demonstrating broadly similar RAAH trends (although French speakers were more likely to not engage in RAAH). Specifically, 15.2% had engaged in one category of RAAH activity, 11.3% in two types, 9.0% in three types, and 6.2% in all four types. Among those who had engaged in RAAH activity (Table 4), physical manipulative activities were most reported (67.5%). Roughly half engaged in herbal/nutritional RAAH (55.1%) and general RAAH activities (49.7%). Alternative belief systems RAAH activities were the least RAAH activities respondents engaged in, with a 42.3% engagement rate. Specific RAAH activities reported as being undertaken by 5% or more of the respondents are listed in Table 5. Some other rare, but serious risk-associated chiropractic practices (e.g., high-velocity and forceful thrust spinal manipulative procedures) were also reported, although used by only 4% of respondents (see dataset for full list of all RAAH activities reported).

**Table 3:** Number of categories of RAAH behaviours reported.

RAAH Categories Engaged In	English (N = 1287)		French (N = 205)		Total (N = 1492)	
	No	%	No	%	No	%
0	734	57.0	136	66.3	870	58.3
1	193	15.0	34	16.6	227	15.2
2	151	11.7	18	8.8	169	11.3
3	122	9.5	12	5.9	134	9.0
4	87	6.8	5	2.4	92	6.2

**Table 4:** Overall RAAH activities reported by category.

	English (N = 553)		French (N = 69)		Total (N = 622)	
Type of RAAH Behaviours	Number	%	Number	%	Number	%
Physical Manipulative RAAH	381	68.9	39	56.5	420	67.1
Herbal/Nutritional RAAH	317	57.3	26	37.7	343	55.1
General RAAH	275	49.7	34	49.3	309	49.7
Alternative Belief Systems RAAH	236	42.7	27	39.1	263	42.3

**Table 5:** Specific RAAH activities reported by 5% or more of respondents.

RAAH Activity		Responses	% (All)	% (RAAH)
**Physical Manipulative RAAH**				
	Cervical spinal manipulative therapies	201	12.6	32.3
**Herbal/Nutritional RAAH**				
	Herbal remedies/supplement in doses much larger than normally orally ingested in your diet	120	7.5	19.2
	Herbal remedies/supplements/pills that contain heavy metals	89	5.6	14.3
	Use of any of alder buckthorn, almond oil, Aloe vera, Angelica, anise, or autumn crocus in pregnancy	78	4.9	12.5
**General RAAH**				
	Used alternative healthcare instead of the existing conventional standard of care for a medically treatable condition	180	11.3	28.9
	Used alternative therapeutics alongside existing medical treatments without informing the medical provider	142	8.9	22.8
	Used therapies based on information provided by alternative healthcare websites, email marketing or social media, or used alternative healthcare for the treatment of a medical condition based on advertising/marketing	133	8.4	21.4
	Used alternative health treatments for conditions diagnosed by alternative practitioners that are not currently recognized as biomedical illnesses. E.g., fatigue, chronic Lyme disease, *Candida* overgrowth, adrenal fatigue, subluxation, food allergies diagnosed without blood/skin prick testing etc.	123	7.7	19.8
	Used alternative therapeutics which were new and where the side effects were unknown or unclear	91	5.7	14.6
	Undertook physically invasive alternative therapeutic procedures. E.g., intravenous therapy or irrigation therapies for colon cleansing not performed by medical doctors or nurses in a hospital setting	72	4.5	11.6
**Alternative Belief Systems RAAH**				
	Traditional Chinese medicine ‐ Acupuncture needling	194	12.2	31.2
	Traditional Chinese medicine ‐ Cupping	109	6.9	17.5
	Naturopathic and homeopathic ‐ Alternative vaccination therapies or vaccine substitutes, such as vitamins or homeopathic vaccines	74	4.6	11.9
	Traditional Chinese medicine ‐ Acupuncture needling with moxibustion/heat	71	4.5	11.4
	Faith healing	67	4.2	10.8
	Naturopathic and homeopathic ‐ Intravenous therapies by naturopaths for vitamin supplementation or chelation	52	3.3	8.4

### Logistic regression

The basic logistic regression analysis produced similar findings as to those obtained by ZINB analysis; therefore, for brevity, only the more comprehensive ZINB analysis is reported here. Exponential ZINB coefficients of demographic variables and of psychological factors can be found in Tables 6 and 7, respectively.

**Table 6:** Exponentiated ZINB coefficients: Demographic associations with RAAH at the 95% CI.

		Count Portion of the Model^a^		Logistic Portion of the Model^b^	
		Unadj. Expected Change in RAAH (95% CI)	Adj. Expected Change in RAAH (95% CI)^c^	UOR (95% CI)	AOR (95% CI)^c^
**Overall RAAH Use**					
	**Gender**				
	Men	Ref	Ref	Ref	Ref
	Women	0.98 (0.77 ‐ 1.24)	**1.42 (1.15 ‐ 1.74)**	0.59 (0.42 ‐ 0.84)	0.97 (0.68 ‐ 1.37)
	Other	1.32 (0.58 ‐ 2.99)	**2.20 (1.12 ‐ 4.33)**	0.53 (0.14 ‐ 2.07)	1.34 (0.37 ‐ 4.92)
	**Income**				
	Below $10,000	Ref		Ref	Ref
	$10,000 ‐ 24,999	0.99 (0.55 ‐ 1.80)		0.49 (0.23 ‐ 1.04)	**0.43 (0.22 ‐ 0.87)**
	$50,000 ‐ 74,999	0.89 (0.51 ‐ 1.55)		0.35 (0.17 ‐ 0.73)	**0.32 (0.16 ‐ 0.63)**
	$75,000 ‐ 99,999	1.08 (0.61 ‐ 1.92)		0.56 (0.28 ‐ 1.13)	**0.47 (0.23 ‐ 0.95)**
	$100,000 ‐ 124,999	0.78 (0.43 ‐ 1.42)		0.20 (0.07 ‐ 0.57)	**0.21 (0.09 ‐ 0.48)**
	$125,000 ‐ 149,999	1.19 (0.59 ‐ 2.40)		0.43 (0.16 ‐ 1.11)	**0.35 (0.13 ‐ 0.91)**
	$150,000 or more	1.34 (0.68 ‐ 2.63)		0.39 (0.15 ‐ 0.98)	**0.27 (0.10 ‐ 0.72)**
	**Chronic Illness**				
	No	Ref	Ref	Ref	Ref
	Yes	1.33 (1.06 ‐ 1.66)	1.18 (0.98 ‐ 1.44)	0.27 (0.17 ‐ 0.42)	**0.26 (0.18 ‐ 0.38)**
	**Healthcare Professional**				
	Does not work in healthcare	Ref		Ref	Ref
	Works in healthcare field	1.38 (1.06 ‐ 1.80)		0.40 (0.23 ‐ 0.70)	**0.49 (0.31 ‐ 0.78)**
	**Age**				
	34 and below	Ref	Ref	Ref	Ref
	35 ‐ 54	0.75 (0.59 ‐ 0.96)	0.96 (0.78 ‐ 1.18)	0.62 (0.43 ‐ 0.91)	0.93 (0.64 ‐ 1.35)
	55 years and above	0.58 (0.41 ‐ 0.83)	0.83 (0.60 ‐ 1.14)	1.30 (0.83 ‐ 2.04)	**2.03 (1.22 ‐ 3.39)**
	**Ethnicity**				
	Caucasian	Ref		Ref	Ref
	Asian	Ref (0.69 ‐ 1.44)		2.26 (1.41 ‐ 3.62)	**2.14 (1.37 ‐ 3.35)**
**General RAAH**					
	**Chronic Illness**				
	No	Ref		Ref	Ref
	Yes	1.04 (0.87 ‐ 1.25)		0.34 (0.26 ‐ 0.46)	**0.31 (0.23 ‐ 0.43)**
	**Healthcare Professional**				
	Does not work in healthcare	Ref		Ref	Ref
	Works in healthcare field	1.24 (1.02 ‐ 1.52)		0.59 (0.42 ‐ 0.84)	**0.65 (0.44 ‐ 0.95)**
	**Age**				
	34 and below	Ref	Ref	Ref	Ref
	35 ‐ 54	0.79 (0.66 ‐ 0.95)	0.92 (0.77 ‐ 1.11)	0.99 (0.73 ‐ 1.33)	0.97 (0.69 ‐ 1.37)
	55 years and above	0.80 (0.60 ‐ 1.07)	1.04 (0.76 ‐ 1.41)	1.94 (1.25 ‐ 3.02)	**2.05 (1.25 ‐ 3.36)**
	**Ethnicity**				
	Caucasian	Ref		Ref	Ref
	Asian	1.42 (1.08 ‐ 1.86)		2.07 (1.30 ‐ 3.31)	**1.99 (1.20 ‐ 3.30)**
**Alternative Belief Systems RAAH**					
	**Gender**				
	Men	Ref	Ref	Ref	Ref
	Women	0.57 (0.40 ‐ 0.82)		0.46 (0.31 ‐ 0.67)	**0.68 (0.47 ‐ 0.99)**
	**Income**				
	Below $10,000	Ref	Ref	Ref	Ref
	$50,000 ‐ 74,999	1.59 (0.50 ‐ 5.03)	**2.46 (1.25 ‐ 4.85)**	0.47 (0.09 ‐ 2.44)	
	$75,000 ‐ 99,999	1.44 (0.45 ‐ 4.60)	**2.41 (1.23 ‐ 4.72)**	0.30 (0.04 ‐ 2.25)	
	$100,000 ‐ 124,999	0.90 (0.26 ‐ 3.04)	**2.07 (1.01 ‐ 4.26)**	-	
	$125,000 ‐ 149,999	1.83 (0.43 ‐ 7.75)	**2.64 (1.15 ‐ 6.05)**	0.55 (0.07 ‐ 4.48)	
	$150,000 or more	2.95 (0.69 ‐ 12.62)	**2.54 (1.14 ‐ 5.65)**	0.93 (0.16 ‐ 5.45)	
	**Chronic illness**				
	No	Ref		Ref	Ref
	Yes	1.30 (0.91 ‐ 1.86)		0.43 (0.29 ‐ 0.62)	**0.36 (0.26 ‐ 0.51)**
	**Education**				
	High school	Ref		Ref	Ref
	College	0.69 (0.39 ‐ 1.24)		0.61 (0.35 ‐ 1.06)	0.77 (0.46 ‐ 1.28)
	Bachelor and above	0.68 (0.40 ‐ 1.16)		0.40 (0.24 ‐ 0.68)	**0.44 (0.27 ‐ 0.71)**
**Physical Manipulative RAAH**					
	**Healthcare Professional**				
	Does not work in healthcare	Ref	Ref	Ref	
	Works in healthcare field	1.74 (1.18 ‐ 2.57)	**1.65 (1.25 ‐ 2.18)**	0.69 (0.38 ‐ 1.25)	
	Other				
	**Income**				
	$50,000 ‐ 74,999	2.73 (0.37 ‐ 20.15)		0.77 (0.04 ‐ 13.86)	**0.27 (0.08 ‐ 0.91)**
	$75,000 ‐ 99,999	3.26 (0.44 ‐ 24.27)		1.19 (0.07 ‐ 21.48)	0.32 (0.09 ‐ 1.14)
	$100,000 ‐ 124,999	2.87 (0.38 ‐ 21.46)		0.33 (0.02 ‐ 6.91)	**0.08 (0.02 ‐ 0.35)**
	$125,000 ‐ 149,999	2.13 (0.26 ‐ 17.57)		0.19 (0.00 ‐ 10.0)	**0.09 (0.02 ‐ 0.47)**
	$150,000 or more	5.20 (0.69 ‐ 39.15)		0.78 (0.04 ‐ 14.56)	**0.13 (0.03 ‐ 0.60)**
	**Chronic illness**				
	No	Ref		Ref	Ref
	Yes	1.10 (0.74 ‐ 1.65)		0.26 (0.13 ‐ 0.55)	**0.21 (0.12 ‐ 0.39)**
	**Education**				
	High school	Ref	Ref	Ref	Ref
	College	0.72 (0.38 ‐ 1.35)	0.62 (0.36 ‐ 1.07)	0.36 (0.15 ‐ 0.83)	**0.29 (0.11 ‐ 0.76)**
	Bachelor and above	0.88 (0.49 ‐ 1.57)	0.75 (0.45 ‐ 1.24)	0.32 (0.15 ‐ 0.68)	**0.22 (0.09 ‐ 0.56)**
	**Ethnicity**				
	Caucasian	Ref		Ref	Ref
	Asian	1.14 (0.52 ‐ 2.50)		5.22 (2.06 ‐ 13.22)	**4.56 (2.09 ‐ 9.94)**
**Herbal/Nutritional RAAH**					
	**Income**				
	Below $10,000	Ref	Ref	Ref	Ref
	$50,000 ‐ 74,999	1.17 (0.52 ‐ 2.61)	1.85 (0.94 ‐ 3.66)	0.72 (0.29 ‐ 1.78)	
	$75,000 ‐ 99,999	1.24 (0.54 ‐ 2.84)	2.02 (0.98 ‐ 4.15)	0.94 (0.37 ‐ 2.38)	
	$100,000 ‐ 124,999	0.88 (0.36 ‐ 2.13)	2.00 (0.95 ‐ 4.20)	0.47 (0.17 ‐ 1.32)	
	$125,000 ‐ 149,999	1.70 (0.69 ‐ 4.20)	**2.17 (1.00 ‐ 4.70)**	0.57 (0.20 ‐ 1.65)	
	$150,000 or more	1.95 (0.73 ‐ 5.20)	**3.10 (1.29 ‐ 7.44)**	1.26 (0.41 ‐ 3.89)	
	**Chronic illness**				
	No	Ref		Ref	Ref
	Yes	0.93 (0.67 ‐ 1.31)		0.38 (0.25 ‐ 0.56)	**0.41 (0.28 ‐ 0.59)**
	**Education**				
	High school	Ref		Ref	Ref
	College	0.84 (0.52 ‐ 1.38)		0.70 (0.40 ‐ 1.22)	0.72 (0.43 ‐ 1.22)
	Bachelor and above	0.89 (0.56 ‐ 1.41)		0.75 (0.44 ‐ 1.27)	**0.55 (0.33 ‐ 0.93)**
	**Age**				
	34 and below	Ref	Ref	Ref	Ref
	35 ‐ 54	0.90 (0.63 ‐ 1.28)	1.14 (0.83 ‐ 1.55)	0.73 (0.48 ‐ 1.12)	0.79 (0.51 ‐ 1.24)
	55 years and above	1.00 (0.56 ‐ 1.79)	1.29 (0.72 ‐ 2.33)	2.36 (1.27 ‐ 4.40)	**2.72 (1.35 ‐ 5.48)**
	**Ethnicity**				
	Caucasian	Ref		Ref	Ref
	Asian	0.91 (0.49 ‐ 1.71)		2.14 (1.09 ‐ 4.17)	**2.49 (1.38 ‐ 4.50)**

AOR = Adjusted Odds Ratio, UOR = Unadjusted Odds Ratio

a Count component ‐ full model ‐ models the number of RAAH behaviours respondents engaged in.

b Logistic component- zero model ‐ models respondents non-engagement in RAAH behaviours.

c Note, variables with no adjusted measures of association were excluded from the final model and hence absent.

**Table 7:** Exponentiated ZINB Coefficients: Psychometric instruments and engagement with RAAH at the 95% CI.

		Count Portion of the Model		Logistic Portion of the Model	
		Unadj. Expected Change in RAAH (95% CI)	Adj. Expected Change in RAAH (95% CI)	UOR (95% CI)	AOR (95% CI)
**Total**					
**Overall RAAH** ^**a**^					
	StP-II-B	1.69 (1.49 ‐ 1.90)	**1.88 (1.63 ‐ 2.16)**	1.21 (0.99 ‐ 1.47)	1.14 (0.86 ‐ 1.49)
	RBAS	1.08 (0.90 ‐ 1.29)	0.99 (0.84 ‐ 1.18)	1.08 (0.80 ‐ 1.47)	1.03 (0.75 ‐ 1.40)
	SOM	0.99 (0.91 ‐ 1.08)	0.98 (0.90 ‐ 1.06)	1.12 (0.97 ‐ 1.29)	1.04 (0.90 ‐ 1.21)
	PAS	0.88 (0.80 ‐ 0.98)	**0.77 (0.69 ‐ 0.85)**	1.35 (1.12 ‐ 1.64)	**1.23 (1.02 ‐ 1.49)**
	CBI	1.21 (1.01 ‐ 1.44)	1.10 (0.91 ‐ 1.34)	1.76 (1.27 ‐ 2.44)	1.21 (0.85 ‐ 1.72)
**General RAAH** ^**b**^					
	StP-II-B	1.44 (1.31 ‐ 1.58)	**1.48 (1.32 ‐ 1.67)**	0.79 (0.64 ‐ 0.97)	**0.70 (0.55 ‐ 0.89)**
	RBAS	1.16 (1.01 ‐ 1.33)	1.04 (0.91 ‐ 1.19)	0.95 (0.73 ‐ 1.23)	0.90 (0.67 ‐ 1.21)
	SOM	1.02 (0.95 ‐ 1.09)	1.03 (0.95 ‐ 1.11)	1.22 (1.09 ‐ 1.38)	1.13 (0.98 ‐ 1.30)
	PAS	0.90 (0.83 ‐ 0.98)	**0.84 (0.78 ‐ 0.91)**	1.40 (1.21 ‐ 1.61)	**1.33 (1.13 ‐ 1.56)**
	CBI	1.16 (1.00 ‐ 1.35)	1.02 (0.85 ‐ 1.22)	1.46 (1.10 ‐ 1.92)	1.02 (0.73 ‐ 1.42)
**Alternative Belief Systems RAAH** ^**c**^					
	StP-II-B	1.93 (1.66 ‐ 2.26)	**2.11 (1.74 ‐ 2.56)**	1.25 (0.99 ‐ 1.59)	1.13 (0.86 ‐ 1.47)
	RBAS	1.20 (0.91 ‐ 1.58)	0.95 (0.75 ‐ 1.21)	1.26 (0.91 ‐ 1.75)	1.18 (0.84 ‐ 1.65)
	SOM	1.01 (0.88 ‐ 1.15)	0.93 (0.83 ‐ 1.04)	1.13 (0.97 ‐ 1.31)	1.02 (0.88 ‐ 1.19)
	PAS	0.89 (0.77 ‐ 1.03)	**0.85 (0.74 ‐ 0.97)**	1.30 (1.09 ‐ 1.56)	**1.31 (1.09 ‐ 1.58)**
	CBI	1.25 (0.96 ‐ 1.64)	0.87 (0.67 ‐ 1.13)	1.46 (1.04 ‐ 2.05)	1.00 (0.69 ‐ 1.44)
**Physical Manipulative RAAH** ^**d**^					
	StP-II-B	1.59 (1.35 ‐ 1.87)	**1.52 (1.26 ‐ 1.84)**	1.86 (1.38 ‐ 2.51)	1.54 (0.99 ‐ 2.41)
	RBAS	1.43 (1.06 ‐ 1.94)	1.16 (0.88 ‐ 1.53)	1.74 (1.12 ‐ 2.71)	1.26 (0.72 ‐ 2.22)
	SOM	0.96 (0.82 ‐ 1.12)	0.91 (0.79 ‐ 1.04)	0.81 (0.64 ‐ 1.02)	**0.75 (0.56 ‐ 1.00)**
	PAS	0.88 (0.73 ‐ 1.06)	**0.82 (0.72 ‐ 0.93)**	1.06 (0.81 ‐ 1.40)	1.00 (0.73 ‐ 1.38)
	CBI	1.57 (1.15 ‐ 2.15)	**1.33 (0.99 ‐ 1.80)**	1.78 (1.13 ‐ 2.80)	1.29 (0.70 ‐ 2.38)
**Herbal/Nutritional RAAH** ^**e**^					
	StP-II-B	1.79 (1.51 ‐ 2.12)	**2.06 (1.61 ‐ 2.64)**	1.10 (0.85 ‐ 1.43)	1.02 (0.72 ‐ 1.45)
	RBAS	1.14 (0.84 ‐ 1.54)	0.95 (0.74 ‐ 1.22)	1.77 (1.21 ‐ 2.59)	**1.79 (1.20 ‐ 2.68)**
	SOM	1.03 (0.91 ‐ 1.16)	0.96 (0.86 ‐ 1.08)	1.18 (1.01 ‐ 1.38)	1.05 (0.88 ‐ 1.25)
	PAS	1.09 (0.91 ‐ 1.30)	**0.81 (0.69 ‐ 0.97)**	1.46 (1.16 ‐ 1.83)	1.21 (0.97 ‐ 1.50)
	CBI	1.36 (1.04 ‐ 1.79)	1.03 (0.77 ‐ 1.37)	1.75 (1.21 ‐ 2.54)	1.35 (0.88 ‐ 2.07)

AOR = Adjusted Odds Ratio, UOR = Unadjusted Odds Ratio

a Count portion of final model adjusted for: age, gender, and chronic illness; logistic portion of final model adjusted for: age, gender, ethnicity, education, income, chronic illness, and healthcare professional.

b Count portion of final model adjusted for: age, and gender; logistic portion of final model adjusted for: age, gender, ethnicity, income, chronic illness, and healthcare professional.

c Count portion of final model adjusted for: age, gender, income, and health status; logistic portion of final model adjusted for: age, gender, education, chronic illness, and healthcare professional.

d Count portion of final model adjusted for: age, gender, education, and healthcare professional; logistic portion of final model adjusted for: age, gender, ethnicity, education, income, health status, and chronic illness.

e Count portion of final model adjusted for: age, gender, employment, and income; logistic portion of final model adjusted for: age, gender, ethnicity, education, and chronic illness.

#### Engagement with RAAH and demographic characteristics

Women respondents, and those who did not identify as men or women, engaged in more RAAH overall activity compared to men, increasing the expected number of RAAH engagement by 1.42 (95% CI = 1.15–1.74) and 2.20 (95% CI = 1.12–4.33), respectively. Across the four RAAH categories, other genders also trended to engage in more RAAH behaviours compared to men, although differences were only statistically significant for those identifying as women in the logistic portion for the alternative belief systems RAAH category (AOR = 0.68; 95% CI = 0.47–0.99).

Older respondents (55 years and above) had significantly higher odds of not engaging in RAAH overall with an AOR of 2.03 (95% CI = 1.22–3.39), and in the general and herbal/nutritional categories as compared to younger respondents (aged 34 years or younger), with AOR of 2.05 (95% CI = 1.25–3.36) and 2.72 (95% CI = 1.35–5.48), respectively.

Respondents with annual income levels of $50,000 or more had significantly higher numbers of RAAH behaviours in the categories of alternative belief systems RAAH and herbal/nutritional RAAH compared to those with income levels below $10,000. The logistic regression portion of the ZINB analysis showed that the odds of non-engagement in RAAH overall and in physical manipulative RAAH were generally significantly lower among respondents with higher income levels, as compared to those with income of less than $10,000.

Respondents who worked in the healthcare field also had significantly higher numbers of RAAH in the physical manipulation category, increasing the expected number of RAAH engaged in by 1.65 (95% CI = 1.25–2.18), as compared to those who do not work in the healthcare field. Healthcare professionals were also more likely to engage in RAAH overall and in general RAAH, with AORs of non-engagement in RAAH of 0.49 (95% CI = 0.31–0.78) and 0.65 (95% CI = 0.44–0.95), respectively.

Similarly, compared to respondents with high school education, those with a bachelor’s degree or higher had significantly lower odds of non-engagement in RAAH for alternative belief systems RAAH, physical manipulative RAAH, and herbal/nutritional RAAH categories, with AORs of 0.44 (95% CI = 0.27–0.71), 0.22 (95% CI = 0.09–0.56), and 0.55 (95% CI = 0.33–0.93), respectively. Having chronic illness was also associated with significantly lower odds of non-engagement in RAAH overall and across all RAAH categories as compared to those with no chronic illness.

Finally, Asian as compared to Caucasian respondents had significantly higher odds of non-engagement in RAAH overall (AOR = 2.14; 95% CI = 1.37–3.35), general RAAH (AOR = 1.99; 95% CI = 1.20–3.30), physical manipulative RAAH (AOR = 4.56; 95% CI = 2.09–9.94), and herbal/nutritional RAAH (AOR = 2.49; 95% CI = 1.38–4.50).

#### Engagement with RAAH and psychometric instruments

Of the five scales, StP-II-B and PAS were the only scales statistically significant and positively associated with overall RAAH engagement and in all four RAAH categories (general RAAH, alternative belief systems RAAH, physical manipulative RAAH and herbal/nutritional RAAH). In the count model a one unit increase in the average StP-II-B score increased the expected number of RAAH behaviours by 1.88 (95% CI = 1.63–2.16), 1.48 (95% CI = 1.32–1.67), 2.11 (95% CI = 1.74–2.56), 1.52 (95% CI = 1.26–1.84), and 2.06 (95% CI = 1.61–2.64), respectively. The logistic portion of the model also illustrated a negative association with non-engagement in general RAAH category (AOR = 0.70; 95% CI = 0.55–0.89). An analysis of the sub-factors of the StP-II-B scale is illustrated in Table 8.

**Table 8:** Adjusted and unadjusted ZINB regression results of RAAH engagement by StP‐II-B factors.

		Count Portion of the Model	Logistic Portion of the Model
		Adj. Expected Change in RAAH (95% CI)	AOR (95% CI)
**Overall RAAH**			
	Premeditation	1.03 (0.96 ‐ 1.11)	1.03 (0.89 ‐ 1.18)
	Consistency	1.00 (0.92 ‐ 1.09)	0.90 (0.76 ‐ 1.06)
	Novelty	**1.10 (1.02 ‐ 1.19)**	0.93 (0.82 ‐ 1.07)
	Self-control	1.03 (0.96 ‐ 1.10)	**0.87 (0.77 ‐ 0.99)**
	Social influence	1.07 (0.99 ‐ 1.17)	1.06 (0.91 ‐ 1.23)
	Similarity	0.98 (0.91 ‐ 1.05)	1.05 (0.91 ‐ 1.21)
	Risk preference	**1.15 (1.07 ‐ 1.22)**	0.97 (0.87 ‐ 1.09)
	Attitudes towards advertising	**1.09 (1.01 ‐ 1.18)**	1.20 (1.03 ‐ 1.40)
	Need for cognition	1.01 (0.93 ‐ 1.10)	**1.18 (1.02 ‐ 1.38)**
	Need for uniqueness	1.05 (0.97 ‐ 1.14)	0.89 (0.76 ‐ 1.05)
**General RAAH**			
	Premeditation	1.05 (0.98 ‐ 1.13)	1.04 (0.91 ‐ 1.19)
	Consistency	0.99 (0.91 ‐ 1.08)	0.88 (0.75 ‐ 1.03)
	Novelty	1.07 (0.99 ‐ 1.16)	**0.86 (0.76 ‐ 0.97)**
	Self-control	1.00 (0.93 ‐ 1.06)	0.95 (0.84 ‐ 1.07)
	Social influence	**1.09 (1.01 ‐ 1.18)**	1.09 (0.94 ‐ 1.25)
	Similarity	1.02 (0.95 ‐ 1.09)	0.92 (0.80 ‐ 1.05)
	Risk preference	1.05 (0.99 ‐ 1.11)	**0.82 (0.73 ‐ 0.91)**
	Attitudes towards advertising	1.06 (0.98 ‐ 1.15)	1.13 (0.99 ‐ 1.29)
	Need for cognition	1.03 (0.96 ‐ 1.11)	1.04 (0.90 ‐ 1.20)
	Need for uniqueness	0.99 (0.92 ‐ 1.07)	**0.83 (0.72 ‐ 0.96)**
**Alternative Belief Systems RAAH**			
	Premeditation	1.01 (0.90 ‐ 1.12)	1.08 (0.93 ‐ 1.25)
	Consistency	**0.89 (0.79 ‐ 0.99)**	**0.84 (0.70 ‐ 1.00)**
	Novelty	**1.17 (1.05 ‐ 1.30)**	1.02 (0.89 ‐ 1.17)
	Self-control	1.01 (0.92 ‐ 1.11)	0.94 (0.82 ‐ 1.07)
	Social influence	**1.15 (1.04 ‐ 1.28)**	1.02 (0.88 ‐ 1.19)
	Similarity	0.98 (0.89 ‐ 1.08)	1.05 (0.91 ‐ 1.22)
	Risk preference	**1.14 (1.05 ‐ 1.24)**	0.98 (0.87 ‐ 1.11)
	Attitudes towards advertising	**1.25 (1.11 ‐ 1.41)**	1.08 (0.93 ‐ 1.27)
	Need for cognition	0.98 (0.87 ‐ 1.09)	1.13 (0.96 ‐ 1.32)
	Need for uniqueness	0.98 (0.87 ‐ 1.11)	0.97 (0.82 ‐ 1.14)
**Physical Manipulative RAAH**			
	Premeditation	1.05 (0.90 ‐ 1.21)	1.17 (0.86 ‐ 1.57)
	Consistency	1.07 (0.91 ‐ 1.26)	1.04 (0.73 ‐ 1.49)
	Novelty	0.93 (0.81 ‐ 1.08)	0.75 (0.55 ‐ 1.03)
	Self-control	**1.14 (1.00 ‐ 1.30)**	1.00 (0.75 ‐ 1.32)
	Social influence	1.00 (0.86 ‐ 1.17)	1.06 (0.77 ‐ 1.46)
	Similarity	1.05 (0.94 ‐ 1.18)	1.01 (0.78 ‐ 1.31)
	Risk preference	**1.22 (1.09 ‐ 1.36)**	1.18 (0.95 ‐ 1.46)
	Attitudes towards advertising	0.97 (0.84 ‐ 1.11)	1.01 (0.73 ‐ 1.38)
	Need for cognition	0.89 (0.76 ‐ 1.05)	1.04 (0.74 ‐ 1.44)
	Need for uniqueness	**1.20 (1.04 ‐ 1.39)**	1.26 (0.87 ‐ 1.83)
**Herbal/Nutritional RAAH**			
	Premeditation	**1.15 (1.03 ‐ 1.29)**	1.01 (0.86 ‐ 1.20)
	Consistency	1.00 (0.88 ‐ 1.13)	0.92 (0.75 ‐ 1.12)
	Novelty	**1.11 (1.00 ‐ 1.24)**	0.92 (0.78 ‐ 1.07)
	Self-control	0.97 (0.88 ‐ 1.07)	**0.86 (0.74 ‐ 1.00)**
	Social influence	1.02 (0.90 ‐ 1.15)	0.99 (0.83 ‐ 1.18)
	Similarity	1.00 (0.90 ‐ 1.11)	0.87 (0.74 ‐ 1.04)
	Risk preference	**1.12 (1.03 ‐ 1.21)**	1.00 (0.87 ‐ 1.14)
	Attitudes towards advertising	1.03 (0.92 ‐ 1.16)	1.09 (0.91 ‐ 1.30)
	Need for cognition	1.09 (0.97 ‐ 1.22)	1.13 (0.95 ‐ 1.35)
	Need for uniqueness	1.04 (0.92 ‐ 1.18)	0.91 (0.75 ‐ 1.11)

The PAS scale was negatively associated with overall and all RAAH categories, in which a one unit increase in the average PAS score decreased the expected number of RAAH risk behaviours in the count portion of the model by 0.77 (95% CI = 0.69–0.85), 0.84 (95% CI = 0.78–0.91), 0.85 (95% CI = 0.74–0.97), 0.82 (95% CI = 0.72–0.93), and 0.81 (95% CI = 0.69–0.97), respectively. The logistic portion of the models also demonstrated significant positive associations for the PAS scales for the non-engagement in overall RAAH, general RAAH, and alternative belief systems RAAH.

The only other scales with significant associations with engagement in RAAH were the CBI in the physical manipulative RAAH category and the RBAS in the herbal/nutritional RAAH category. Apart from a positive association with physical manipulative RAAH in the count model (expected number of RAAH increase by 1.33 (95% CI = 0.99–1.80) for a one unit change in the mean CBI score), the CBI scale was also positively associated, but not significantly, with number of overall RAAH, general RAAH, herbal/nutritional RAAH. The RBAS was positively associated with non-engagement in herbal/nutritional RAAH in the logistic model (AOR = 1.79; 95% CI = 1.20–2.68). Similarly, the SOM scale was negatively associated with non-engagement in physical manipulative RAAH with a borderline statistical significance AOR of 0.75 (95% CI = 0.56–1.00).

The SOM and RBAS scales were negatively associated with the overall number of AH, alternative belief systems RAAH, and herbal/nutritional RAAH engaged in by respondents in the count portion. However, these associations were again, not statistically significant.

## Discussion

### Respondent characteristics

In this study, RAAH uptake varied by respondent characteristics, including gender, age, education, income, ethnicity, and having chronic illnesses. The factors that were found to influence engagement in AH were also associated with frequency of engaging in some RAAH behaviours previously identified as moderate to high risk (Table 6).

Women, those who did not identify gender, and individuals with higher level of education and higher incomes were more likely to engage in AH (Table 6), thus replicating findings in prior work [52, 53]. Women are more frequent users of AH and suggested reasons for this include women often being the primary caregivers in families and having greater health needs [53–57]. Interestingly, although previous work suggests older people may suffer more harm from AH [22], respondents over 55 were over twice as likely not to engage in AH, general or herbal/nutritional RAAH practices. This may reflect increasing risk aversity with age and that much AH advertising is targeted at younger people and adolescents; however, findings on this vary [2, 9, 38, 58–60].

In terms of income, as with prior research, more affluent people tended to use AH and engage in RAAH activities more, especially physical manipulative RAAH (Table 6). Health expenditures have been positively related to the prevalence of overall and physical AH treatments [2, 61]. This finding reflects the fact that most AH is commercially provided and costly, and likely that the single most reported use of AH is for back pain for which costly chiropractic treatment is a common option [62–64].

Education is another demographic factor associated with AH uptake. Several studies have indicated that those with higher education are more frequent users of AH [9, 61, 65], as was revealed here. However, researchers have also found that respondents with lower education and health literacy levels are more likely to believe health misinformation [66]. Overall, there may be a more nuanced relationship between level and type of education (e.g., arts- versus science-based) and income and other demographics at play here, making further exploration of this phenomenon of interest.

Chronic illness was also a pertinent factor in RAAH uptake, and findings from the ZINB analysis were consistent with logistics regression results in this respect. Chronic illness has been identified as a significant factor in the use of AH in a number of studies, as these individuals often find existing biomedical care not meeting their needs [4, 67, 68]. Those who reported poor health status were also more likely to seek treatment using alternative systems of belief, such as naturopathy or traditional Chinese medicine, possibly as a result of a desire to try alternative frameworks to scientific biomedicine.

Those Canadians who reported being of Asian ethnicity were about twice as likely to not engage in AH and in general or herbal/nutritional RAAH practices, and over four times less likely to engage in physical manipulative RAAH. This finding contrasts with some studies that reported more medical pluralism [68–70], although others have also identified lower uptake of chiropractic amongst Asians, Hispanics Blacks and Native Americans than Whites [71–74]. The reasons for this are unclear, although one study suggests disclosure of AH use may be lower in Asian populations [68], and others indicate socio-economic factors correlate with chiropractic use [71, 72, 74, 75]. This is likely as chiropractic is a costly therapy and represented the least uptake, while herbal and supplement use is cheaper. However, most earlier studies are over a decade old and from outside of Canada.

Intriguingly, the survey also showed that individuals working in the healthcare field were 50% more likely to engage in RAAH as compared to those who did not. This finding seems contrary to what might be expected. A possible explanation for this finding could be the perception of healthcare professionals that they are better able to weigh the risks and advertised benefits of engaging in any form AH, or that these individuals have more interest in exploring therapeutic options. Such assessments may not be common among individuals who have more limited knowledge about AH. Additionally, the survey did not require respondents to identify their profession, thus some of these responses may have been from AH professionals.

### RAAH behaviours

Among those who engaged in any form of AH, physical manipulative therapies and herbal/ nutritional supplements were the most common types of RAAH reported, with 68% and 55%, respectively (Tables 3 and 4). In particular, use of chiropractic cervical manipulation was reported by 13% of all respondents. The more serious risk-associated chiropractic practices (although rare and used by only 4% of respondents) are also noteworthy. Chiropractic is a well-established and widely used form of AH in Canada; however, there remains considerable controversy over risks associated with some of the interventions marketed by this profession, such as cervical spinal manipulation [2, 16, 76–79]. It appears those undertaking these therapies are either unaware of the potentially serious side effects (including arterial dissection and stroke) or are more risk tolerant.

The wide range of providers and marketing of herbal/nutritional supplements makes them easily accessible as well as the reported poor regulation of advertising standards with regard to nutritional supplements may explain their widespread uptake [80]. The activities reported included some RAAH behaviours of more serious concern, such as the use of supplements in high doses and of those with known toxicity (Table 5), suggesting current regulation of advertising and supplement sales is somewhat ineffective in this domain.

Following this, general RAAH practices were reported by about half of those respondents using AH (Table 4). Some reported using AH instead of medical treatment (12% of all respondents), using AH alongside medical treatment without informing their doctor (10%), using AH treatments advertised (9%) or using AH for conditions not medically recognized (8%) and with unknown side effects (6%). Additionally, 5% of all respondents indicated they had used invasive procedures (such as intravenous therapies or enemas) from AH providers in settings outside of hospitals (Table 5). These findings confirm the widespread use of AH, although these RAAH activities are less commonly engaged in.

As other researchers have suggested [81, 82], this confirms many AH users (29%) are using AH instead of medical treatment, rather than as complementary to it, and many of them are reluctant to discuss their AH use with their physician (23%). More concerningly, at least one in ten users of AH engaged in high-risk invasive or untested procedures. Again, this suggests that although less common, RAAH behaviours are relatively widespread and around 5% of people are highly risk-tolerant in this area and prepared to undertake risky therapies with serious negative potential outcomes compared to any known benefits. The impact of personality factors and relatively unrestricted advertising of healthcare likely both influence this behaviour.

The RAAH activities involving alternative belief systems were least utilized by the respondents (42%). These RAAH activities mainly consisted of using acupuncture and cupping from traditional Chinese medicine practitioners, with 31% and 18% of AH users, respectively. These practices are widespread in Canada and even promoted by some doctors and athletes; however, they remain controversial in terms of both efficacy and safety [19, 83–85]. Other higher risk-associated behaviours in this group were reported by 5% of all respondents (Table 5). Although rarer, some of these, such as naturopathic intravenous therapy, are of particularly higher risk [2, 86–89]. The use of naturopathic alternatives to vaccines also poses significant risks to the community, including increased vaccination hesitancy and reduced protection against serious contagions [90–92].

### The value of psychometric tools in predicting engagement

People who engaged in RAAH demonstrated significantly different StP-II-B and PAS outcome scores to those who did not, and those who engaged in more RAAH demonstrated StP-II-B and PAS outcome scores significantly different than those who engaged in them less. However, the null-hypothesis was not rejected for the CBI, RBAS, and SOM instruments.

The StP-II-B was most significant instrument for predicting overall RAAH engagement, with estimates showing between one and a half and twice as much increase in the numbers of RAAH for a unit increase in average StP-II-B scores. Of note, risk preference (tolerance) was the most significant sub-factor as a predictor in all categories (Tables 6 and 7). This finding reflected a significant influence of higher risk tolerance, desire for novelty, social influence, and personal susceptibility to advertising on RAAH uptake (which can be viewed as a form of consumer behaviour). Some of these StP-II-B elements have been previously identified as drivers in AH uptake, and are reflected in the advertising of AH products and services [2, 4, 9, 23, 24, 31]. For example, typical advertising for supplements by naturopaths often emphasizes novelty and the ability to control one’s own health outside of biomedical therapies. Such advertising frequently references positive social role models such as professionals, celebrities, athletes or “moms.” [38, 93, 94] Additionally, AH practitioners have also been demonstrated as being more active on social media where advertising is less regulated [95].

The PAS scale was also associated with engaging in RAAH overall and by RAAH category (Table 7). Those who scored higher in the PAS (suggesting more trust in science) were associated with less engagement in RAAH. These findings appear to confirm the principle that a negative attitude toward and mistrust of biomedical science is associated with increased engagement in AH, and with being more risk-averse with RAAH therapies [8, 96]. Some researchers have noted trust in scientists during the recent pandemic has been a key factor behind individual support for public health initiatives and vaccination policy, although social media has been used quite successfully by antivaccination and by some AH advocates to counter this [10, 97–99]. One recent study also suggested public trust in the effectiveness of AH therapies is not mutually exclusive with a belief in science, due to disinformation and the negative impact of *big pharma* scandals [100]. However, claims of a scientific basis of RAAH in terms of prescription, communication, and marketing may play a significant role in determining trust in them for many. This has been observed in many AH domains where pseudoscience is used in their marketing, and is a demonstrated effective advertising strategy [6, 101, 102].

The CBI was marginally significant associated with engagement in frequency of physical manipulation, but not with other types of RAAH (Table 7). On the other hand, the RBAS (apart from herbal/nutritional RAAH) and SOM were generally not associated with either engagement in AH or frequency of engagement in RAAH. Hence, here respondents’ desire for self-control, satisfaction with public medical provision or their desire to seek out positive rewards all had less effect on engagement with RAAH and risk tolerance. However, the desire for a rapid solution/reward may be more of a motivator for some to try more risky herbal remedies or supplements. In the case of RAAH, the suggested negative motivator of experiencing less empathic practitioners [103], or unpleasant public health systems encouraging people to try AH, may not be such a significant factor overall [104]. Nevertheless, it does seem to be a motivator for physical manipulative RAAH. This may reflect the current lack of effective medical treatments (or those with unpleasant side effects) in the management of chronic musculoskeletal pain.

### Limitations

This study is the largest and most comprehensive study to investigate AH and risk in Canada. The sample used in this study is in most respects representative of the Canadian adult population, and thus these results can help to inform public debate relating to the uptake of RAAH. The fact that some responses were contradictory may indicate that some questions were not fully understood by all respondents (e.g., due to low health literacy), and the study used self-reported engagement in RAAH, which may be influenced by competing values–and is common when issues are morally complex. Furthermore, since the survey was directed at adults, this work does not address RAAH use in children. Another limitation is that the order in which survey questions are presented is known to influence individuals’ responses, as preceding questions may provide context for those that follow.

## Conclusions

RAAH uptake in the Canadian public was influenced by the characteristics of gender, age, income, education, employment, chronic illness status, and ethnicity. Engagement in some form of RAAH was not unusual (around 40%). The most common types of RAAH use reported were physical manipulation and herbal/nutritional supplement use, although other higher-risk AH activities (including use of toxins and physically invasive procedures) were also reported by around 5% of respondents. The StP-II-B and PAS instruments were predictive of the likelihood of engagement in RAAH behaviours (illustrated by higher risk tolerance, desire for novelty, positive attitude to advertising and social influence), while the CBI, RBAS and SOM psychometric instruments were not overall. The CBI and SOM instruments were predictive of engagement with physical manipulative RAAH and the RBAS was predictive of herbal/nutritional RAAH engagement. This study identifies that uptake of RAAH is a significant health concern in Canadian public health and illustrates the need to advocate for evidence-based policy and practice. Understanding how to best identify and educate the public on the significant risks encountered with some AH therapeutics is an important part of health promotion.

## Supporting information

S1 File Survey instrument. (PDF)
