# Supplementary material for: Demographic and psychometric predictors associated with engagement in risk-associated alternative healthcare behaviours
Source: PLoS One. 2023 Sep 21;18(9):e0291016. doi: 10.1371/journal.pone.0291016 (PMC10513319; doi:10.1371/journal.pone.0291016)
Supplement: S1 File — (PDF) [file pone.0291016.s001.pdf]

Risk-Taking Behaviours with Alternative Healthcare

Default Question Block

Risk-Taking Behaviours with Alternative Healthcare Survey

Principal Investigators:

1. Dr. Bernie Garrett,
Associate Professor
School of Nursing,
University of British Columbia,

2. Timothy Caulfield
Professor
Health Law Institute, Faculty of Law
University of Alberta

Introduction to Survey & Consent Information

As a part of a research project to explore the nature of alternative healthcare we would appreciate you filling in an online questionnaire about your experiences with alternative healthcare products and services in Canada.

Purpose of the Study

This study is aimed at exploring the experiences of residents of Canada with alternative healthcare, and the factors that encourage people to use it. This work is being carried out by researchers from the University of British Columbia, School of Nursing and the University of Alberta, Health Law Institute.

Study Procedures

We are looking for study participants who are residents of Canada and are at least 16 years old.

You are being asked to participate in a short web-survey and your participation in this study will require the following.

Completion of an online questionnaire, consisting of a range of questions asking for your experiences of and thoughts about alternative health products and services. Some biographical information is asked for in the survey, but the survey does not require your name, and all the survey data will be stored and analyzed anonymously. It is estimated the questionnaire will take approximately 15 minutes to complete.

Compensation

We thank you in advance for your time in considering this invitation and participating in this study. As a token of appreciation for your participation all respondents to the online survey will have the opportunity to be entered into a prize draw for two $200 Amazon Gift Voucher prizes.

If you would like to be entered in the draw for the $200 Amazon Gift Voucher prize, please enter your email at the end of the survey. This email will not be associated with your responses in any analysis and is simply required so we may enter you into the draw. It will be removed from your responses before we analyze them. Any participants who withdraw from the study will remain in the draw.

CONSENT

Rights as a Participant

Your participation in this project is entirely voluntary and you may refuse to participate or withdraw from the project or any element of the activities listed above at any time. However, once the survey has been submitted it may not be possible to remove your data from the analysis as it will have been entered anonymously into our analysis spreadsheets.

Research Use and Confidentiality

By agreeing to participate in this project, you will be allowing the research team to use and analyze the materials you produce. Although some biographical information will be required for the questionnaire this will be kept securely and anonymously in all documentation used during the analysis of the data. All physical documents and files pertaining to this study will be identified only by code number only and kept in a locked filing cabinet in a locked office in the UBC School of Nursing. Any data kept on electronic media (computers) at UBC's School of Nursing will not include the name or personal details of the individual subject and will be encrypted, kept solely on the computers of the principal researcher, Dr. Garrett. As a research participant, you will not be identified by name in any reports of the completed study. There are no known risks to participating in any aspect of this research program.

Contact for information about the study

If you have any questions related to this project or wish to have further information with respect to the study, you may contact the Principal Investigator at (+1) 604-875 8203. bernie.garrett@nursing.ubc.ca

Contact for concerns about the rights of research subjects

If you have any concerns or complaints about your rights as a research participant and/or your experiences while participating in this study, contact the Research Participant Complaint Line in the UBC Office of Research Ethics at 604-822-8598 or if long distance e-mail RSIL@ors.ubc.ca or call toll free 1-877-822-8598.

Default Question Block

Note: The definition of several medical terms and health practices can be displayed by tapping or hovering your cursor over the underlined words in the questions. They may take a few seconds to appear.

1. How old are you?

- ○ 16-19 years old
- ○ 20-24 years old
- ○ 25-34 years old
- ○ 35-44 years old
- ○ 45-54 years old
- ○ 55-64 years old
- ○ 65-74 years old
- ○ 75 years or more

2. Gender: How do you identify?

- ○ Man
- ○ Non-binary
- ○ Woman
- ○ Prefer to self-describe ______
- ○ Prefer not to say

3. What is the highest degree or level of school you have completed? If currently enrolled, highest degree received.

- ○ No schooling completed
- ○ Nursery school to 8th grade
- ○ Some high school, no diploma
- ○ High school graduate, diploma or the equivalent (for example: GED)
- ○ Some college credits, no degree
- ○ Trade/technical/vocational training
- ○ Associate degree
- ○ Bachelor's degree

- ○ Master's degree
- ○ Doctorate degree

4. Do you work in the healthcare field?

- ○ Yes
- ○ No

5. How would you describe your ethnic background/origins?

- ○ North American Aboriginal Origin
- ○ Caucasian / European
- ○ Asian / Pacific Islander
- ○ Hispanic / Latino
- ○ Black or African American
- ○ Other (please specify) ______

6. Employment Status: Are you currently?

- ☐ Employed for wages (part-time or full-time)
- ☐ Self-employed
- ☐ Retired
- ☐ A homemaker
- ☐ A student
- ☐ Out of work
- ☐ Unable to work

7. In which of the following groups does your annual total personal income fall?

- ○ Less than $10,000
- ○ $10,000 - $24,999
- ○ $25,000 - $49,999
- ○ $50,000 - $74,999
- ○ $75,000 - $99,999
- ○ $100,000 - $124,999
- ○ $125,000 - $149,999
- ○ $150,000 or more

8. Overall, how would you categorize your health status?

- ○ Very healthy
- ○ Generally healthy
- ○ Frequently unwell

9. Do you have any chronic illness?

- ○ Yes
- ○ No

10. Have you ever used alternative health care therapies or services (i.e. those that are distinct from conventional medicine)?

- ○ Yes
- ○ No

11. Which of the following alternative health care activities have you engaged in?

- ☐ Generally, using alternative health care remedies or therapies instead of or alongside conventional medical treatments
- ☐ Activities involving specific alternative health belief frameworks such as traditional Chinese medicine, homeopathy, naturopathy, etc.
- ☐ Alternative physical manipulation therapies or interventions, e.g. chiropractic, massage or osteopathic therapies
- ☐ Alternative herbal or nutritional interventions including those regulated as natural health products, e.g. herbal supplements, essential oils and other products not formally regulated as drugs

Risky alternative health care behaviours

12. Did you do any of the following when you engaged in the use of these alternative healthcare products and services?

A) General category

	Yes	No
1. Used alternative healthcare instead of the existing conventional standard of care for a medically treatable condition	○	○
2. Used alternative therapeutics which were new and where the side effects were unknown or unclear	○	○
3. Undertook physically invasive alternative therapeutic procedures, e.g. intravenous therapy or irrigation therapies for colon cleansing not performed by medical doctors or nurses in a hospital setting	○	○
4. Used alternative therapeutics alongside existing medical treatments without informing the medical provider	○	○
5. Used therapies based on information provided by alternative healthcare websites, email marketing or social media, or used alternative healthcare for the treatment of a medical condition based on advertising/marketing	○	○
6. Used alternative health treatments for conditions diagnosed by alternative practitioners that are not currently recognized as biomedical illnesses. E.g., fatigue, chronic Lyme disease, Candida overgrowth, adrenal fatigue, subluxation, food allergies diagnosed without blood/skin prick testing etc.	○	○

B1) Have you used any of the following traditional Chinese medicine (TCM) remedies?

	Yes	No
1. Monkshood (Aconitum napellus)	○	○
2. Thunder God Vine (Tripterygium Wilfordii)	○	○
3. Jia Yi Jian capsules or tea (also known as Chinese Viagra)	○	○
4. Asarum, Bragantia (Apama or Thottea)	○	○
5. Niuhuang Jiedu Pian	○	○
6. Bak Foong Pills	○	○
7. Fabao nurturing hair tonic	○	○
8. Cupping	○	○
9. Acupuncture needling	○	○
10. Acupuncture needling with moxibustion/heat	○	○

B2) Have you used any of these naturopathic and homeopathic therapies?

	Yes	No
1. Intravenous therapies by naturopaths for vitamin supplementation or chelation	○	○
2. Naturopathic colonic irrigation therapies for colon cleansing	○	○
3. Alternative vaccination therapies or vaccine substitutes, such as vitamins or homeopathic vaccines	○	○

B3) Have you used any of these Ayurvedic medicinal products?

	Yes	No
1. Guggul tablets	○	○
2. Sundari Kalp	○	○

	Yes	No
3. Jambrulin	○	○

C) Use of religious health advice or faith healing:

	Yes	No
1. Have you ever taken religious or spiritual advice that conflicted with health service providers' advice (e.g. religious advice about reproductive health that conflicted with medical advice)?	○	○
2. Used faith healing	○	○

D1) have you used any of these chiropractic therapies?

	Yes	No
1. Cervical (neck vertebrae) spinal manipulative therapies (SMT)	○	○
2. Forceful SMT procedures such as Atlas Orthogonal Technique, Activator, Diversified, Koren Specific Technique or hammer and chisel technique	○	○
3. Used chiropractic care as an alternative to vaccination	○	○
4. High-velocity thrust SMT with a pre-existing musculoskeletal problem with instability of joints	○	○

D2) Have you ever used this osteopathic therapy?

	Yes	No
Prolotherapy injections	○	○

E) To your knowledge, have you used any of these herbal natural remedies?

	Yes	No
1. Herbal remedies/supplements/pills that contain metals like aluminum, silver, lead, mercury, tin, and zinc (e.g., Crude Tan Pills, Crude Red Pills, Precious Pills for women, Yang Chun Kou Fu Ye, Reinforce Sex Pill, Crocodile Bile Pills, Wuchi Pai Feng Wan / Wu Ji Bai Feng Wan etc.)	○	○
2. Herbal remedies/supplements with unlisted pharmacologic ingredients like arsenic, betamethasone, diazepam (e.g., Wau Wa cream, Muijiza cream, Tung Shueh pills etc.)	○	○
3. Herbal remedies/supplement in doses much larger than normally orally ingested in your diet	○	○
4. Weight-loss aids containing nitrosofenfluramine (an analogue of fenfluramine) or chromium picolinate (e.g., Slim 10, Arsenal X etc.)	○	○
5. Use of any of Alder buckthorn, Almond oil, Aloe vera, Angelica, Anise, or Autumn crocus in pregnancy	○	○
6. Use of Miracle Mineral Solution (MMS - a chlorine dioxide solution) therapy	○	○

Persuasion

Please indicate the strength of your agreement with the following statements:

13. Premeditation

	Strongly disagree	Disagree	Somewhat disagree	Neither agree or disagree	Somewhat agree	Agree	Strongly agree
I only act to satisfy immediate concerns, figuring the future will take care of itself.	○	○	○	○	○	○	○
I think that sacrificing now is usually unnecessary since future outcomes can be dealt with at a later time.	○	○	○	○	○	○	○
I only act to satisfy immediate concerns, figuring that I will take care of future problems that may occur at a later date.	○	○	○	○	○	○	○

14. Consistency

	Strongly disagree	Disagree	Somewhat disagree	Neither agree or disagree	Somewhat agree	Agree	Strongly agree
The appearance of consistency is an important part of the image I present to the world.	O	O	O	O	O	O	O
An important requirement for any friend of mine is personal consistency.	O	O	O	O	O	O	O
I make an effort to appear consistent to others.	O	O	O	O	O	O	O

15. Novelty

	Strongly disagree	Disagree	Somewhat disagree	Neither disagree or agree	Somewhat agree	Agree	Strongly agree
I would like to travel to places that are strange and far away.	O	O	O	O	O	O	O
I would have enjoyed being one of the first explorers of an unknown land.	O	O	O	O	O	O	O
If it were possible to visit another planet or the moon for free, I would be among the first to sign up.	O	O	O	O	O	O	O

16. Self-control

	Strongly disagree	Disagree	Somewhat disagree	Neither disagree or agree	Somewhat agree	Agree	Strongly agree
I say inappropriate things.	O	O	O	O	O	O	O
I do certain things that are bad for me, if they are fun.	O	O	O	O	O	O	O
Sometimes I can't stop myself from doing something, even if I know it is wrong.	O	O	O	O	O	O	O

17. Social Influence

	Strongly disagree	Disagree	Somewhat disagree	Neither disagree or agree	Somewhat agree	Agree	Strongly agree
When buying products, I generally purchase those brands that I think others will approve of.	O	O	O	O	O	O	O
If other people can see me using a product, I often purchase the brand they expect me to buy.	O	O	O	O	O	O	O
I achieve a sense of belonging by purchasing the same products and brands that others purchase.	O	O	O	O	O	O	O

18. Similarity

	Strongly disagree	Disagree	Somewhat disagree	Neither disagree or agree	Somewhat agree	Agree	Strongly agree
When a product I own becomes popular among the general population, I begin to use it less.	O	O	O	O	O	O	O
I often try to avoid products or brands that I know are bought by the general population.	O	O	O	O	O	O	O
The more commonplace a product or brand is among the general population, the less interested I am in buying it.	O	O	O	O	O	O	O

19. Risk Preferences

I would not be worried by:

	Strongly disagree	Disagree	Somewhat disagree	Neither disagree or agree	Somewhat agree	Agree	Strongly agree
Betting a day's income at the horse races	O	O	O	O	O	O	O
Betting a day's income at a high-stake poker game	O	O	O	O	O	O	O
Betting a day's income on the outcome of a sporting event	O	O	O	O	O	O	O

20. Advertising

	Strongly disagree	Disagree	Somewhat disagree	Neither disagree or agree	Somewhat agree	Agree	Strongly agree
Advertising is essential.	O	O	O	O	O	O	O
Advertising helps raise our standard of living.	O	O	O	O	O	O	O
Advertising results in better products for the public.	O	O	O	O	O	O	O

21. Cognition

	Strongly disagree	Disagree	Somewhat disagree	Neither disagree or agree	Somewhat agree	Agree	Strongly agree
I would rather do something that requires little thought than something that is sure to challenge my thinking abilities.	O	O	O	O	O	O	O
I try to anticipate and avoid situations where there is a likely chance I will have to think in depth about something.	O	O	O	O	O	O	O
Learning new ways to think doesn't excite me very much.	O	O	O	O	O	O	O

22. Uniqueness of Choices

	Strongly disagree	Disagree	Somewhat disagree	Neither disagree or agree	Somewhat agree	Agree	Strongly agree
I often combine possessions in such a way that I create a personal image that cannot be duplicated.	O	O	O	O	O	O	O
I often try to find a more interesting version of run-of-the-mill products because I enjoy being original.	O	O	O	O	O	O	O
Having an eye for products that are interesting and unusual assists me in establishing a distinctive image.	O	O	O	O	O	O	O

4. Motivational factors

Please indicate the strength of your agreement with the following statements:

23. Response to reward

	Very true for me	Somewhat true for me	Somewhat false for me	Very false for me
When I'm doing well at something, I love to keep at it.	O	O	O	O
When I get something I want, I feel excited and energized.	O	O	O	O
When I see an opportunity for something I like, I get excited right away.	O	O	O	O
When good things happen to me, it affects me strongly.	O	O	O	O
It would excite me to win a contest.	O	O	O	O

Beliefs

Please indicate the strength of your agreement with the following statements:

24. Satisfaction with conventional medicine

	Strongly disagree	Disagree	Somewhat disagree	Neither agree nor disagree	Somewhat agree	Agree	Strongly agree
Do you think your general practitioner is concerned with your well-being?	O	O	O	O	O	O	O
Do you think your general practitioner's treatment is effective?	O	O	O	O	O	O	O
Do you feel your general practitioner listens to what you say?	O	O	O	O	O	O	O
Do you feel your general practitioner gives you enough time?	O	O	O	O	O	O	O
Do you believe that general practitioners can almost always help their patients feel better?	O	O	O	O	O	O	O

25. At your last visit to your general practitioner, how satisfied were you with your treatment?

Very dissatisfied	Dissatisfied	Somewhat dissatisfied	Neither satisfied or dissatisfied	Somewhat satisfied	Satisfied	Very satisfied
○	○	○	○	○	○	○

Personal Beliefs About Science

Please indicate the strength of your agreement with the following statements:

26. Personal Beliefs

	Strongly disagree	Disagree	Somewhat disagree	Neither agree nor disagree	Somewhat agree	Agree	Strongly agree
Medicine is a science and should be based on rigorous scientific principles.	○	○	○	○	○	○	○
Treatments which are not based on modern scientific discoveries are worthless.	○	○	○	○	○	○	○
Every treatment should be thoroughly tested by doctors and scientists before people are allowed to try it.	○	○	○	○	○	○	○
Complementary therapies should be scientifically evaluated.	○	○	○	○	○	○	○

Control Beliefs

Please read each statement carefully and answer according to how much you agree with each statement by selecting the appropriate answer.

27. Mastery/Self-efficacy

	Strongly disagree	Disagree	Mildly disagree	Mildly agree	Agree	Strongly agree
I know that I can do what is necessary to improve my health.	○	○	○	○	○	○
I am confident that I can successfully look after my health.	○	○	○	○	○	○
Even though there are things I can do to improve my health, I don't feel that I can do them.	○	○	○	○	○	○
I am able to meet the challenge of following a healthy routine.	○	○	○	○	○	○
When facing a health problem, I often feel overwhelmed about what to do.	○	○	○	○	○	○
I am confident that I could deal with any unexpected health problems.	○	○	○	○	○	○
I am confident in my ability to make the right decisions about my health.	○	○	○	○	○	○
When it comes to my health, I often feel unable to do what I know should be done.	○	○	○	○	○	○

28. General Control

	Strongly disagree	Disagree	Mildly disagree	Mildly agree	Agree	Strongly agree
It is my own actions that determine how healthy I am.	○	○	○	○	○	○
If I set my mind to it I can improve my health.	○	○	○	○	○	○
My health depends on how I take care of myself.	○	○	○	○	○	○
People who take care of themselves stay healthy.	○	○	○	○	○	○
How soon I recover from an illness depends on how I look after myself.	○	○	○	○	○	○
My current state of health is a reflection of how I look after myself.	○	○	○	○	○	○
I am certain that with effort I can improve my health.	○	○	○	○	○	○

29. Chance Control

	Strongly disagree	Disagree	Mildly disagree	Mildly agree	Agree	Strongly agree
If I am lucky I will stay healthy.	○	○	○	○	○	○

	Strongly disagree	Disagree	Mildly disagree	Mildly agree	Agree	Strongly agree
My health depends on forces beyond my control.	O	O	O	O	O	O
How soon I recover from an illness depends on how lucky I am.	O	O	O	O	O	O
If I am fortunate my health will improve.	O	O	O	O	O	O
My health is determined by circumstances beyond my control.	O	O	O	O	O	O

30. Symptom Control

	Strongly disagree	Disagree	Mildly disagree	Mildly agree	Agree	Strongly agree
I can take control of my health by managing my day-to-day symptoms.	O	O	O	O	O	O
If I make the effort, I can manage my illness.	O	O	O	O	O	O
There are things that I can do to make my health problem easier to deal with.	O	O	O	O	O	O
I believe that I can do more to control my symptoms.	O	O	O	O	O	O
If I do the right things, I can make my symptoms more manageable.	O	O	O	O	O	O
Regardless of circumstances, there are things I can do to improve my health.	O	O	O	O	O	O

Any feedback on the survey or comments you wish to add?

Draw

If you would like to be entered in the draw for the $200 Amazon Gift Voucher prize, please enter your email below. This email will not be associated with your responses in any analysis and is simply required so we may enter you into the draw. It will be removed from your responses before we analyze them. Any participants who withdraw from the study will remain in the draw.

E-Mail Address for Draw Participation:

Submitting the survey

CONSENT TO PARTICIPATE IN THE STUDY IS INDICATED BY COMPLETION AND SUBMISSION OF THE ONLINE QUESTIONNAIRE.

STUDY ETHICAL REVIEW ID NUMBER H19- 01790

Version #1.1 15/06/2021

Powered by Qualtrics
